# Impact of Three Different Light Spectra on the Yield, Morphology and Growth Trajectory of Three Different *Cannabis sativa* L. Strains

**DOI:** 10.3390/plants10091866

**Published:** 2021-09-09

**Authors:** Philipp Reichel, Sebastian Munz, Jens Hartung, Achim Präger, Stiina Kotiranta, Lisa Burgel, Torsten Schober, Simone Graeff-Hönninger

**Affiliations:** 1 Cropping Systems and Modelling, Institute of Crop Science, University of Hohenheim, 70599 Stuttgart, Germany; s.munz@uni-hohenheim.de (S.M.); achim.praeger@uni-hohenheim.de (A.P.); lisa.burgel@uni-hohenheim.de (L.B.); torsten.schober@uni-hohenheim.de (T.S.); simone.graeff@uni-hohenheim.de (S.G.-H.); 2 Biostatistics, Institute of Crop Science, University of Hohenheim, 70599 Stuttgart, Germany; jens.hartung@uni-hohenheim.de; 3 Department of Agricultural Sciences, Viikki Plant Science Centre, University of Helsinki, P.O. Box 27, FI-00014 Helsinki, Finland; Stiina.kotiranta@helsinki.fi

**Keywords:** *Cannabis sativa*, morphology, growth trajectory, LED, cultivation system

## Abstract

Cannabis is one of the oldest cultivated plants, but plant breeding and cultivation are restricted by country specific regulations. Plant growth, morphology and metabolism can be manipulated by changing light quality and intensity. Three morphologically different strains were grown under three different light spectra with three real light repetitions. Light dispersion was included into the statistical evaluation. The light spectra considered had an influence on the morphology of the plant, especially the height. Here, the shade avoidance induced by the lower R:FR ratio under the ceramic metal halide lamp (CHD) was of particular interest. The sugar leaves seemed to be of elementary importance in the last growth phase for yield composition. Furthermore, the last four weeks of flowering were crucial to influence the yield composition of *Cannabis sativa* L. through light spectra. The dry flower yield was significantly higher under both LED treatments compared to the conventional CHD light source. Our results indicate that the plant morphology can be artificially manipulated by the choice of light treatment to create shorter plants with more lateral branches which seem to be beneficial for yield development. Furthermore, the choice of cultivar has to be taken into account when interpreting results of light studies, as *Cannabis sativa* L. subspecies and thus bred strains highly differ in their phenotypic characteristics.

## 1. Introduction

*Cannabis sativa* L. is one of the oldest crops [1], which despite its multifunctionality [2] has spent much of the last century in illegality as it possesses psychotropic properties [3]. Nowadays it has experienced a certain renaissance and the basis for its legalization for medical purposes is being established slowly all over the world [4,5]. This has led to new, rapidly growing markets in North America and Europe [6]. To ensure the needed high quality standards for medical and pharmaceutical products, it is necessary to scientifically develop and evaluate cultivation methods. Recently, there has been an increased focus on arbuscular mycorrhiza [7], cultivation substrates [8,9], plant density [10], phytohormones [11] and the effect of fungal pathogens [12] to further develop cultivation systems.

In contrast to other agricultural crops, the development of *Cannabis sativa* L. strains was carried out by small breeders which kept the breeding process confidential. This has led to an inflationary availability of strains, which differ morphologically, phenologically and in their overall growth requirements. In addition, comparability between each strain is difficult, because genetic pedigrees are only slowly established [13,14,15] and even if it is stated, the claim about the genetic origin might be incorrect [16,17]. The most commonly agreed upon formal taxonomy is that the genus *Cannabis* comprises one species, *C. sativa* L., with its polymorphic subspecies *sativa*, *indica*, and *ruderalis.* These subspecies highly differ in their phenotypic characteristics and chemical profiles [18,19,20,21]. As interbreeding has led to an opaque blending of different strains, the overall reaction of a specific strain to different cultivation practices might lead to some surprises, if the strain is not well known to the grower and new strains are introduced into a cultivation program.

*Cannabis sativa* L. is exposed to various management and environmental stimuli during its growth, such as propagation by cuttings [22], fertilization [23,24] and different light spectra and quantity [25,26,27] that tend to maximize flower yield meeting at the same time a desired phytochemical profile [28]. Currently, little information is available on whether these stimuli have an effect on plant morphology features such as height, number of branches and their respective length, as well as the overall biomass composition [29]. 

Besides yield, light in particular influences plant morphology, mixing red and blue light caused shorter internodes, smaller leaf area and more compact morphology compared to a pure white light source [30]. A significant increase in yield and concentration of total Δ^9^-tetrahydrocannabinol (THC) was reported when using intra-canopy red and blue lighting compared to the sunlight control treatment [31]. Light as the driving force of photosynthesis has an impact on plant growth and development [32]. Furthermore, specific properties of light sources can have a positive effect on growth and yield [6]. High pressure sodium lamps (HPS) and light emitting diode (LED) are most commonly used as light systems in horticulture [33,34]. In particular, LEDs are seen as a potential replacement for HPS lamps [35] as they have a low heat emission in combination with an increased energy efficiency and a longer operating time [36]. In addition, LEDs allow the spectral quality to be easily varied and individually adapted for each crop [37].

Light research in cannabis has focussed on maximizing flower yield and increasing cannabinoid concentrations. Comparing one strain under three light spectra, a clear effect on morphology, but no yield increase was reported [38]. In another study, two strains were grown under three different light spectra, and the spectrum was changed between the short and long day growing period of the cannabis plants [39]. A significant yield loss was observed under the LED lights, while the strains showed similar light-induced morphological changes. On the other hand, [40] pointed out that no yield difference existed for the tested strain between normal full-spectral LEDs and special horticultural LEDs. Investigating the influence of a light gradient based on one light spectrum and one strain, it was shown that an increase in light intensity can increase flower yield [41]. In contrast to other light studies, [31] applied additional sub canopy lightning and achieved a yield increase, for the considered strain. Recently, [27] tested three strains under four light spectra and pointed out that strains can react differently to light spectra. However, each of the mentioned recent publications used different strains, light sources, and methodological approaches which make the comparability and thus an overall conclusion on the impact of light on cannabis morphology and yield difficult.

The aim of this study was to test the morphological plasticity of different strains under differing light spectra, to provide an understanding of plant growth and yield composition and to investigate whether strains react differently to similar light spectra. Overall, the study will contribute to better understand the influence of light on *Cannabis sativa* L. and help to define target morphological parameters for future cannabis breeding.

## 2. Results

### 2.1. Plant Architecture

**Figure 1:**
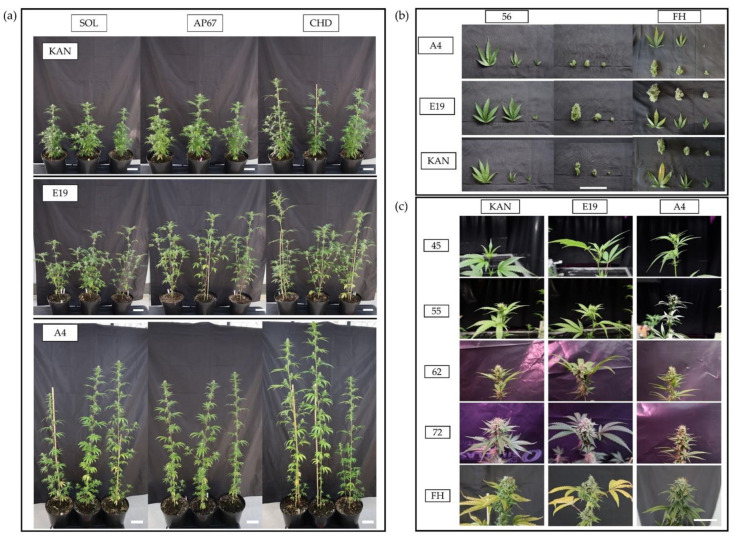
(**a**) The strains KAN, E19 and A4 at 56 DAP under the respective light spectra (scale = 10 cm). The three considered light spectra were CHD Agro 400, AP67 and Solray385. In the following sections, these treatments are abbreviated as CHD, AP67 and SOL. (**b**) Comparison of the considered traits of the individual strains under CHD, 56 DAP and at final harvest (FH). For the leaves, a distinction was made between main leaves, branch leaves and sugar leaves. Flowers were separated at harvest into main bud, top side bud and side bud (scale = 5 cm). (**c**) Course of flower growth of the main bud under AP67 over the trial period of the strains examined (scale = 5 cm).

Differences in architecture were found between each individual strain (Figure 1). The strain Kanada (KAN) expressed a bushy architecture and was in general the shortest. Intermediate height was observed for E19, a strain with a very heterogeneous morphological character, which was shaped by long side branches. The considerably tallest strain was A4 having only few and short branches. With regard to the plant organs, there were clear differences in the expression of the individual leaf morphology. The main leaves (ML) showed variations in the shape of the leaflets at comparable sizes, in which A4 had the narrowest leaflets, followed by KAN and E19. The branch leaves (BL) displayed more distinct differences in their expression with E19 exhibiting the largest in this particular fraction even comparable in size to the main leaves. All strains had a comparable size within the sugar leaves (SL). Across all leaf fractions, a size gradient was evident in the following order ML > BL > SL.

At the time of 56 days after planting (DAP) differences within the three flower fractions became apparent, which were subdivided into main top bud (MTB), side top bud (STB) and side bud (SB). The strains KAN and E19 showed a gradient related to the flower size in the following order MTB > STB > SB throughout the trial. This gradient was also observed for A4 at the time of the final harvest. Besides the flower size, the compactness of the flowers also differentiated between the individual strains. Especially E19 showed a compact flower structure, whereas KAN and E19 displayed a rather loose cluster. All strains revealed flower development 15 days after induction of the short day. Furthermore, the largest allocation of flower mass occurred between 62 and 72 DAP.

#### 2.1.1. Node Number and Length

**Figure 2:**
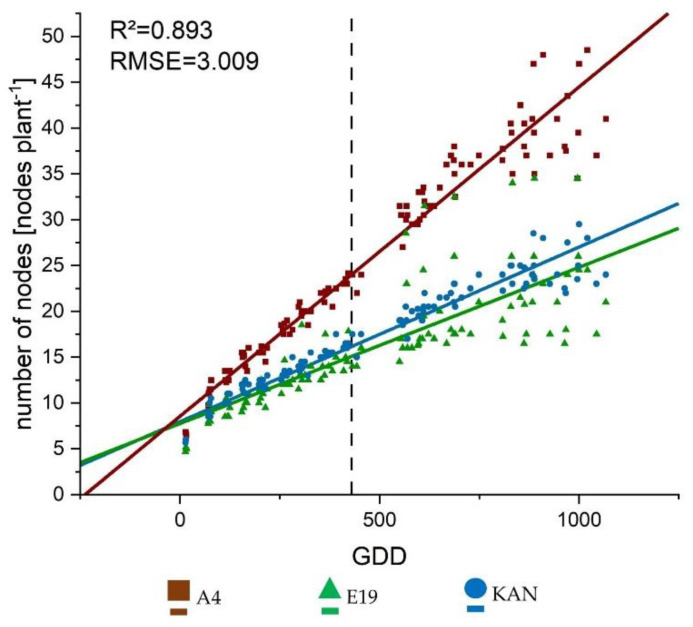
Growth curves fitted to the data collected from the weekly surveys for number of nodes per plant using the linear Equation (3). The lines display the calculated growth trajectory, the respective symbols stand for the mean values of the respective treatment per growing degree day (GDD). The vertical dashed line represents the conversion from long to short day.

**Figure A1:**
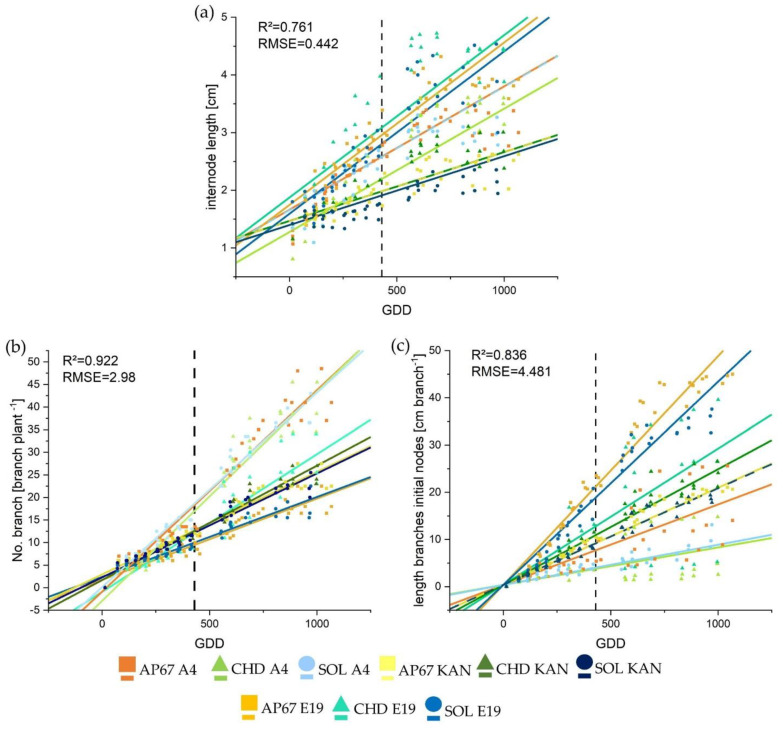
Growth curves fitted to the data collected from the weekly surveys for (**a**) internode length (**b**) number of branches (**c**) length of the branches emerged from the initial nodes after propagation using the linear Equation (3). The lines display the calculated growth trajectory, the respective symbols stand for the mean values of the respective treatment per growing degree day (GDD), when two lines overlap, one is shown as a solid line and the other as a dashed line. The vertical dashed line represents the conversion from long to short day.

The three considered light spectra were CHD Agro 400, AP67 and Solray385. In the following sections, these treatments are abbreviated as CHD, AP67 and SOL. The growth curves represented the linear development processes well showing a R^2^ of 0.893 and 0.761 for number of nodes (Figure 2) and internode length (Figure A1a), respectively. 

For the number of nodes, there was no influence of the light spectra but a clear difference between the strains. Thus, A4 showed a significantly larger slope and an associated nodal formation compared to KAN and E19 (Table 1). The length development of the internodes showed an interaction between the strain and light spectrum in the intercept. By contrast, the slope, differed significantly only between strains with E19 developing significantly faster compared to A4 and KAN (Table 2). 

#### 2.1.2. Canopy Height

**Figure 3:**
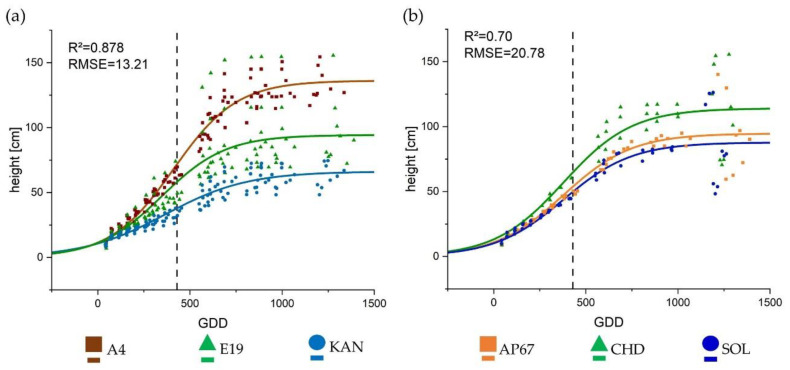
Growth curves fitted to the data collected from the weekly surveys for (**a**) height in [cm] per strain and (**b**) height in [cm] under each respective light using the classical logistic Equation (2). The lines display the calculated growth trajectory, the respective symbols present the mean values of the respective treatment per growing degree day (GDD). The vertical dashed line represents the conversion from long to short day.

The height of the individual plants was based on the number of nodes and the internode distance. The height development continued to approximately 1000 GDD (Figure 3). The development process could be well described by the sigmoidal growth curve achieving an R^2^ of 0.878 and 0.70 for the strain- and light-specific effect, respectively. For height, in contrast to internode elongation, there was no interaction between strain and light. All strains thus behaved the same in the canopy height alteration due to the respective light spectra. Due to the high number of internodes, A4 reached the largest final height of 136.10 cm, followed by E19 (95.25 cm) and KAN which was the significantly shortest strain with 66.11 cm. Under the different light spectra, plants displayed significant differences in their final height ($h_{\max_{p}}$), but no significant differences could be found in relation to light in the other parameters. The plants were more stretched under CHD compared to AP67 and SOL. There were no significant differences between the two LEDs, but a tendency towards shorter plants under SOL was evident (Table 1).

#### 2.1.3. Branch Length and Number

Overall the linear growth trajectory represented well the growth process of branches and the length of the branches emerged from the initial nodes after propagation (Figure A1b,c) showing an R^2^ of 0.922 and 0.836, respectively. Both parameters showed a significant interaction between light and strain. These trajectories clearly underline the different morphological characteristics, as A4 showed the highest increase in the number of branches based on the higher number of nodes, but their growth was significantly lower compared to E19 with the longest branches at final harvest (Table 2). With regards to the interaction between strain and light spectra, no significant differences were found for A4 and KAN except for E19, showing a difference for CHD due to its heterogeneous appearance.

#### 2.1.4. Mean Stem Diameter

**Figure 4:**
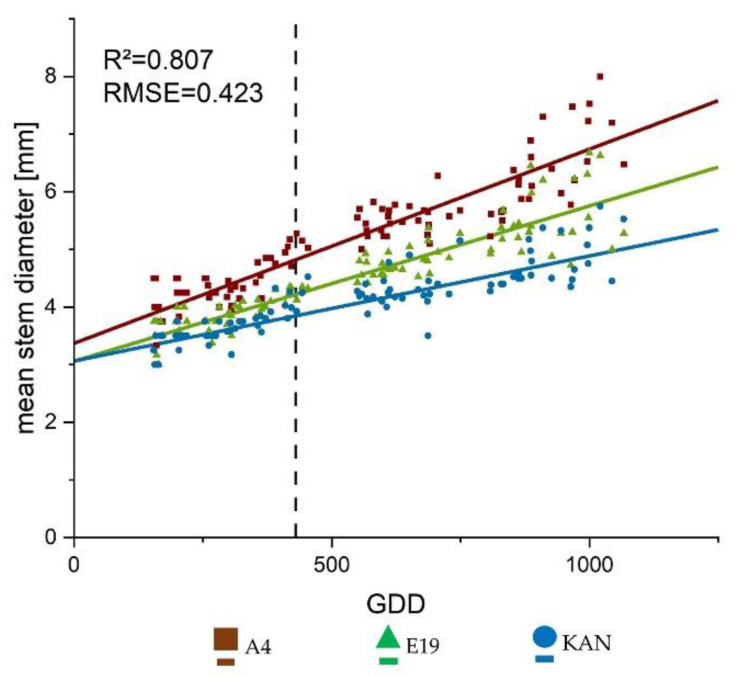
Growth curves fitted to the data collected from the weekly surveys for mean stem diameter using the linear Equation (3). The lines display the calculated growth trajectory, the respective symbols stand for the mean values of the respective treatment per growing degree day (GDD). The vertical dashed line represents the conversion from long to short day.

Overall the linear growth trajectory represented well the diameter growth of the main stem (Figure 4) showing an R^2^ of 0.807. This clearly illustrates the influence of the different heights, as A4 showed the fastest diameter development, followed by E19 and KAN. (Table 1). With regards to the interaction between strain and light spectra, no significant differences were found.

### 2.2. Growth

#### 2.2.1. Dry Matter of Leaves

**Figure 5:**
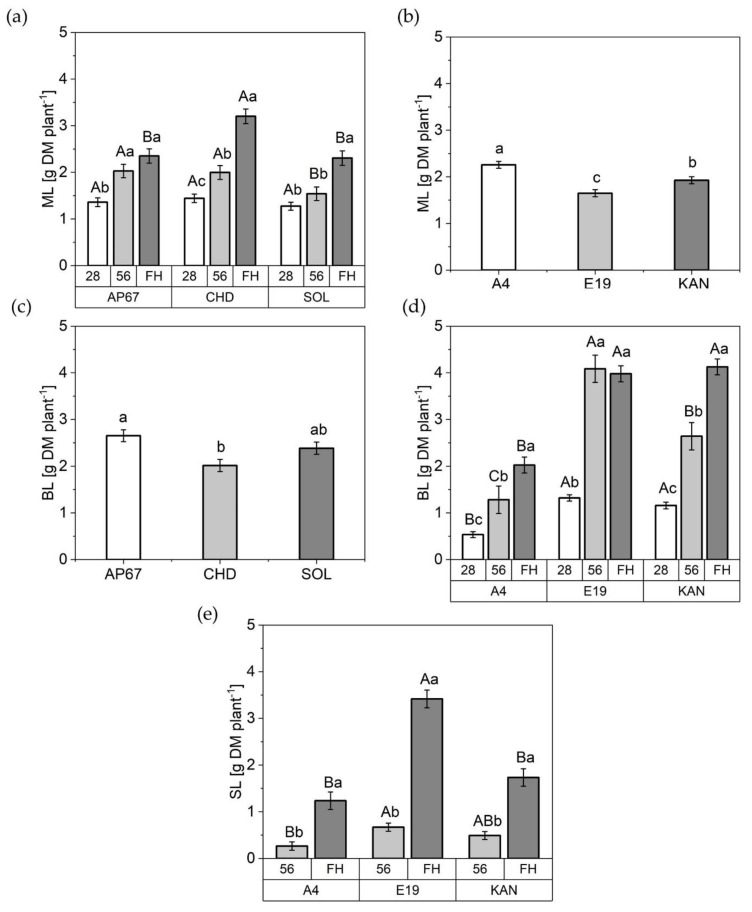
Dry matter (DM) of the different leaf fractions in [g DM plant^−1^]. (**a**) Main leaves (ML) under SOL, AP67, and CHD at 26 DAP, 58 DAP and FH; (**b**) Main leaves (ML) of A4, KAN as well as E19 across all biomass cuts; (**c**) Branch leaves (BL) under AP67, SOL, and CHD across all biomass cuts (**d**) Branch leaves (BL) of A4, KAN and E19 at 28 DAP, 56 DAP and FH. (**e**) Sugar leaves (SL) of A4, KAN and E19 at 56 DAP and final harvest (FH); Means covered by at least one identical lowercase letter did not differ significantly at α = 0.05, within each measurement, strain or light spectra. Means covered by at least one identical uppercase letter did not differ significantly at α = 0.05, within a strain or light spectra as indicated by a Tukey-test.

At the point of final harvest related to the light spectra under CHD the dry matter of the main leaves increased by 40% compared to AP67 (2.35 ± 0.15) and SOL (2.30 ± 0.15) (Figure 5). In regard to the influence of the light over the course of the experiment the main leaves under CHD and SOL displayed a consistently significant increase between 28, 56 days after planting and final harvest. 

At the beginning of the short day at 28 DAP the same mass of main leaves within the light spectra was discovered. In the later plant development, an increase was found under AP67 and CHD of 29% at 56 days after planting compared to SOL. With regards to the branch leaves, there was a significant increase under AP67 with 32% compared to CHD (2.01 ± 0.13). No significant influence of the respective light spectra on the sugar leaves could be detected. 

The strains also displayed significant differences in their leaf formation. A4 formed the most main leaves (2.25 ± 0.07) followed by KAN (1.92 ± 0.07) and E19 (1.64 ± 0.07). In the fraction of branch leaves no significant difference could be found between the strains KAN (4.12 ± 0.16) and E19 (3.97 ± 0.17) however, these were able to form twice the amount compared to A4 at final harvest. Branch leaves of A4 and KAN showed a significant increase between each measurement date, whereas for E19 the maximum growth was already reached at 56 DAP while already exceeding A4 more than three times the mass. Moreover, E19 produced 270% more sugar leaves in comparison to A4 and 200% the amount as KAN. Additionally, between the individual biomass cuts the dry matter of the sugar leaves revealed a significant increase over all strains.

In general, leaf mass allocation increased throughout plant growth in all fractions. With the exception of E19, which showed no increase in branch leaves between 56 DAP and FH.

#### 2.2.2. Specific Leaf Area

**Figure 6:**
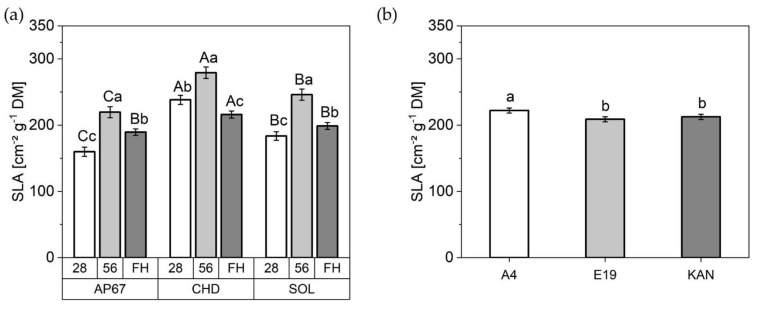
Specific leaf area (SLA) [42] [LA in cm^2^ g^−1^ DM^−1^]: (**a**) Total specific leaf area under AP67, CHD, and SOL at 28 DAP, 56 DAP and FH.; (**b**) SLA of A4, KAN as well as E19 across all biomass cuts. Means covered by at least one identical lower case letter did not differ significantly at α = 0.05, within each measurement or light spectra. Means covered by at least one identical upper case letter did not differ significantly at α = 0.05, within light spectra as indicated by a Tukey-test.

Considering the total specific leaf area, obtained using all specific leaf areas of the individual leaf fractions e.g., main leaves, branch leaves and sugar leaves (Figure 6). There were significant differences already 28 days after planting between each respective light spectrum, with a noticeable increase under CHD (215.96 ± 8.66) compared to SOL (198.78 ± 5.15) and AP67 (189.43 ± 4.97). Between the individual biomass cuts (28 DAP, 56 DAP and FH) an increase under all light spectra between 28 DAP and 56 DAP followed by a significant decrease at the time of final harvest could be detected. CHD was able to significantly increase the specific leaf area under all observation periods. Under SOL, the plants formed the lowest SLA, but this evened out with AP67 at the time of the final harvest. The strain A4 (222.15 ± 3.84) exceeded E19 (208.91 ± 3.83) and KAN (212.53 ± 3.85).

#### 2.2.3. Stem Dry Matter

**Figure 7:**
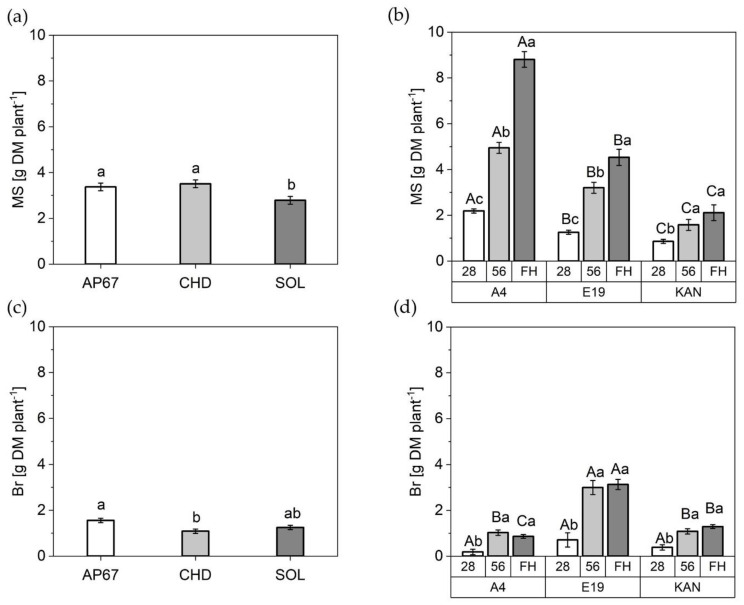
Mean dry matter (DM) of the different stem fractions in [g plant^−1^]: (**a**) Main stem (MS) under AP67, CHD and SOL across all biomass cuts; (**b**) Main stem (MS) of A4, E19, and KAN at 28 DAP, 56 DAP and FH; (**c**) Branches (Br) under AP67, CHD and SOL across all biomass cuts; (**d**) Branches (Br) of A4, E19, and KAN at 28 DAP, 56 DAP and FH. Means covered by at least one identical lowercase letter did not differ significantly at α = 0.05, within each measurement or light spectra. Means covered by at least one identical uppercase letter did not differ significantly at α = 0.05, within strain as indicated by a Tukey-test.

The dry matter of the main stem displayed a significant increase by 21% and 25% under AP67 and CHD respectively compared to SOL (2.78 ± 0.17) (Figure 7). In addition, the mass of the side shoots was increased by 43% under AP67 versus CHD.

Among the strains, A4 (8.08 ± 0.34) with its long and elongated growth pattern showed the highest dry matter of the main stem, followed by E19 (4.53 ± 0.35) and KAN (2.11 ± 0.34). The strain-typical characteristics also became apparent as E19 (3.13 ± 0.22) developed highest branch mass followed by KAN (1.29 ± 0.08) and A4 (0.86 ± 0.08). 

Over the experimental period, the dry matter of the main stem increased steadily until the time of the final harvest, with a significant difference between the respective observation periods. For KAN, a significant increase between 28 DAP and 56 DAP could be observed, but no significant further growth until final harvest. Already at the end of the vegetative period the strains displayed significant differences in relation to main stem, A4 was even able to produce 250% more dry mass of the main stem compared to KAN. The dry matter development of the branches increased significantly between DAP 28 and 56 and then stagnated over all considered strains until the final harvest. 

#### 2.2.4. Photosynthesis Rate

**Figure 8:**
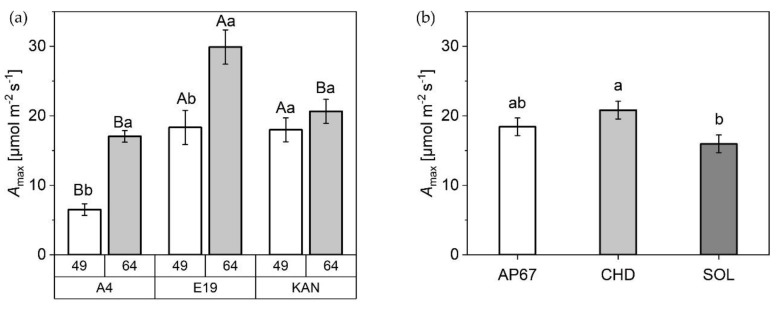
Net photosynthetic capacity in relation to light intensity A_max_ [µmol m^−2^ s^−1^] of (**a**) A4, E19, and KAN at 49 DAP and 64 DAP and (**b**) AP67, CHD and SOL over both acquisition dates. Means covered by at least one identical lowercase letter did not differ significantly at α = 0.05, within each measurement or light spectra. Means covered by at least one identical uppercase letter did not differ significantly at α = 0.05, within strain as indicated by a Tukey-test.

The net photosynthetic capacity (A_max_) under CHD was 30% higher compared to SOL (15.96 ± 1.28) (Figure 8). In relation to the observed strains, E19 (29.98 ± 2.47) showed significantly higher rates of A_max_ compared to KAN (20.64 ± 1.73) and A4 (17.04 ± 0.84) at time 64 DAP. At DAP 49, there were significant differences between KAN (17.98 ± 1.73) and E19 (18.33 ± 2.47) compared to A4 (6.5 ± 0.84). Comparing the two recording times, significant differences can be seen in E19 and A4 between both acquisition dates. No differences could be detected for KAN.

#### 2.2.5. Nitrogen Concentration of the Leaf Fractions

**Figure 9:**
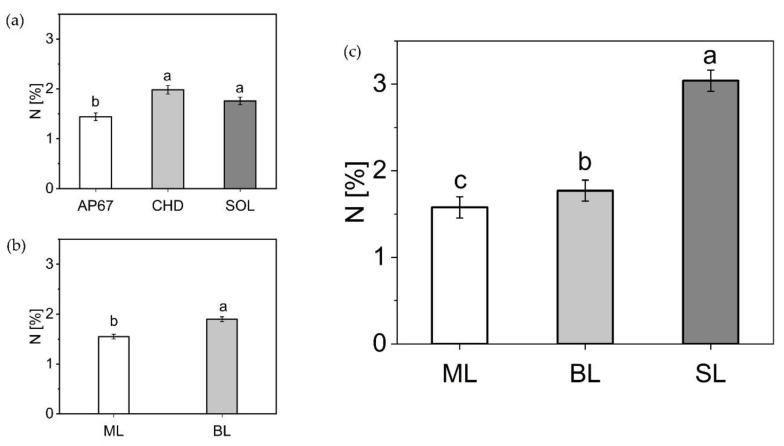
Leaf nitrogen concentration (%). (**a**) All leaf fractions under the respective light spectra at 28 (DAP) (**b**) Main leaves (ML) and branch leaves (BL) at 28 DAP. (**c**) All leaf fractions: main leaves (ML), branch leaves (BL) and sugar leaves (SL) at final harvest (FH). Means covered by at least one identical lowercase letter did not differ significantly at α = 0.05, within each light spectra or leaf fraction as indicated by a Tukey-test.

The first biomass cut showed significant differences between the light spectra (Figure 9), with a higher nitrogen concentration for CHD (1.98 ± 0.08) and SOL (1.76 ± 0.07) compared to AP67 (1.44 ± 0.07). These concentrations were related to the leaf mass formed in the following order CHD < SOL < AP67. 

Among the leaf fractions, there was a significantly higher nitrogen content in the branch leaves (1.9 ± 0.04) compared to the main leaves (1.55 ± 0.04). At the time of the final harvest, significantly higher nitrogen concentrations were found in the sugar leaves (3.04 ± 0.122) of the flower compared to the branch leaves (1.77 ± 0.12) and main leaves (1.57 ± 0.12). At the time of the final harvest, no influence of the light spectra on the nitrogen concentration of the leaf fractions could be detected.

### 2.3. Harvest

The harvest index, the dry yield of the side top buds, the total dry yield and the main top bud demonstrated significant increases across all strains and light spectra between 56 DAP and FH. With regards to the flower fraction side bud, there are significant differences between light spectrum, strain and between the individual biomass cuts (Table 3). The flower yield distribution across all strains was: main top bud 13%, side top bud 43.3% and side bud 43.7%.

**Figure 10:**
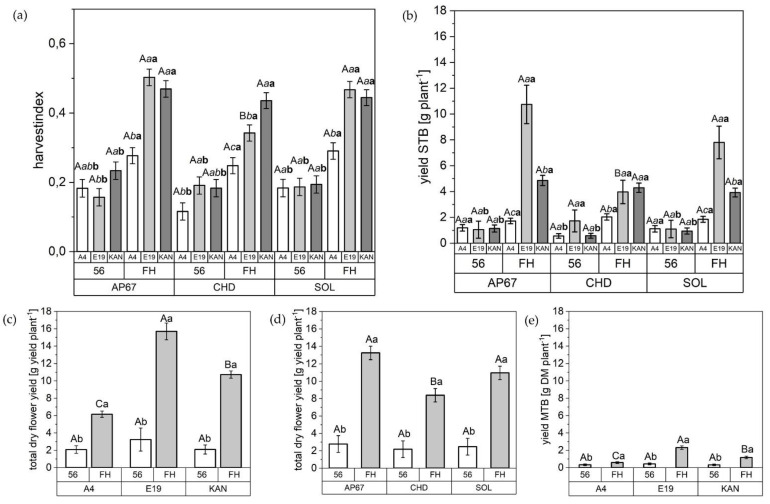
Harvest index [HI]; yield of the individual flower fractions [g DM plant^−1^] and total dry flower yield [g yield plant^−1^] based on the sum of the flower fractions after air drying; (**a**) HI under AP67, CHD and SOL at 56 DAP and FH related to A4, E19, and KAN; (**b**) Yield side top bud (STB) under AP67, CHD and SOL at 56 DAP and FH related to A4, E19, and KAN; (**c**) Total dry flower yield of A4, E19, and KAN at 56 DAP and FH; (**d**) Total dry flower yield under AP67, CHD and SOL at 56 DAP and FH; (**b**) Yield main top bud (MTB) of A4, E19, and KAN at 56 DAP and FH. The following applies to (**a**,**b**): Means covered by at least one identical capital letter did not differ significantly at α = 0.05 within light spectra. Means covered by at least one identical italic and lowercase letter did not differ significantly at α = 0.05, within measurement and means covered by at least one identical bold and lowercase letters did not differ significantly at α = 0.05, within the strains with a specific measurement as indicated by a Tukey-test. For (**c**–**e**) Means covered by at least one identical capital letter did not differ significantly at α = 0.05 within measurement. Means covered covered by at least one identical lowercase letter did not differ significantly at α = 0.05, within a strain or light spectra as indicated by a Tukey-test.

With the exception of E19, there was no difference in the harvest index of the different strains under the respective light spectra at final harvest (Figure 10). For this specific strain, the highest harvest index was found among AP67 (0.50 ± 0.02) and SOL (0.46 ± 0.02), which showed increases of 47% and 35% respectively compared to CHD (0.34 ± 0.02). Additionally, in the development of the experiment, no differences between the strains under the respective light spectra at 56 DAP in relation to the harvest index was observed. 

The KAN strain achieved under AP67 at 56 DAP, a 60% higher harvest index compared to E19 (0.15 ± 0.02). At the time of final harvest, E19 (0.50 ± 0.02) and KAN (0.49 ± 0.02) achieved almost double the harvest index as A4 (0.27 ± 0.02). Looking at SOL, there were no differences between the strains at 56 DAP. Significant differences were only found between E19 (0.46 ± 0.02) and KAN (0.44 ± 0.02) at the time of the final harvest compared to A4 (0.29 ± 0.02). 

The same pattern as for the harvest index was apparent for the flower fraction side top bud. Here, a tendency towards a surplus dry flower yield under AP67 could be observed. However, in this flower fraction no significant difference between the strains at final harvest could be detected, except for E19, where a significant yield reduction under CHD of 260% and 196% compared to AP67 and SOL was achieved. In terms of total dry yield based on all three flower fractions, AP67 (13.23 ± 0.77) was able to achieve the highest yield level across all strains followed by SOL (10.95 ± 0.77) and CHD (8.38 ± 0.77), but no significant difference between AP67 and SOL could be detected.

Significant differences in the total dry flower yield became visible that were typical for the respective strains. For instance, the final harvest of E19, KAN and A4 was 15.69 (±0.96), 10.72 (±0.42) and 6.15 (±0.36). Thus, the yield level of E19 reached 255% and 146% of A4 and KAN, respectively. This was also evident for the main top bud fraction with a significantly larger flower of E19 (2.30 ± 0.20) compared with KAN (1.16 ± 0.14) and A4 (0.58 ± 0.10).

## 3. Discussion

### 3.1. Growth Behavior of the Strains

In this experiment, a cultivation system was defined consisting of a predetermined PAR value of 680 μmol m^−2^s^−1^ (PAR) at a height of 1 m and the same fertilization strategy for all the strains under consideration. Thus, the cultivation system was not adapted to the strain, but the respective strain had to adapt to the predefined cultivation system with its plasticity. Over the trial period, special characteristics of the respective strains became apparent. In relation to the dry mass of the leaf area, A4, which is in its growth patter similar to a fiber hemp strain, produced the highest leaf mass of the main leaves with simultaneously the lowest fraction of branch leaves. The morphology of E19 and KAN was characterized with a higher proportion of branches, wherefore, the ratio of main and branch leaves shifted towards the latter. The strains thus behaved as expected based on their final morphology (Figure 1).

Strikingly, the nitrogen concentration shifted towards the sugar leaves at the end of the flowering phase. This indicated a shift in the importance of the respective leaf fractions throughout the growth. During the long day phase, the main leaves dominated at first and towards the end, a balanced relationship between branch leaves and main leaves emerged. Due to the induction of flowering with the short day [43], the ratio shifted to the branch leaves and with the onset of maturity in which senescence affected branch leaves as well as the main leaves, we detected a significant difference between sugar leaves, branch leaves and main leaves based on their respective nitrogen concentration. This confirms the findings of [44] that especially in the last weeks the sugar leaves of the flower are elementary important for the photosynthetic capacity of the flower. This is highlighted by the significant increase in dry mass of the sugar leaves across all strains from DAP 56 to FH (Figure 5) in combination with the highest N concentration (Figure 9).

In terms of leaf area, specific leaf area increased significantly across all leaf fractions and strains during the development of *Cannabis sativa* L. from the initiation of the short-day period at DAP 28 to DAP 56. This suggests larger leaves at constant weight to possibly supply the increased biomass allocation in the flowering phase with assimilates. This is supported by the fact that during this period photosynthetic efficiency increased significantly across A4 and E19 (Figure 8). The increase in specific leaf area was followed by a significant decrease towards the final harvest. This is a normal behavior as the leaves diminish in diameter as the main stem continues to develop [43] (Figure 4 and Figure 7). Nevertheless, it should be noted that the response of the specific leaf area to nitrogen fertilization can be very dynamic [45]. Therefore, further research on morphology of *Cannabis sativa* L. in relation to fertilizer is needed to define the specific leaf area more precisely.

Based on the harvest index (Figure 10), there is a clear advantage in the morphology of KAN and E19 based on shorter growth and a balanced ratio between main stem and branches (Figure 7). This is supported by a significant increase in dry yield of E19 compared to the other strains, which at the same time showed the highest increase in branch growth (Figure A1a). This finding establishes the groundwork for the use of various pruning techniques to shift the branch/stem ratio towards the development of branches, such as topping, in order to realize an increase in total dry yield [46,47]. The results support the hypothesis that taller plants are quicker to harvest [48], as A4 had the earliest harvest date. However, we cannot confirm the theory of higher productivity of tall plants in terms of yield parameters, as A4 produced the lowest yield.

All parameters were associated with a linear or sigmoid growth that increased and stagnated above 1000 GDD (Figure 2, Figure 3 and Figure 4, and A1), by then 570 GDD had elapsed after flower induction. This indicated that *Cannabis sativa* L. stops growing towards 600 GDD after flower induction and shifts the biomass allocation towards the flowers, which is also confirmed by the strong growth of the yield components in the last DAPs (Figure 10). The number of nodes for the hemp type [49] strain A4 differed significantly compared to KAN and E19. This suggests that based on the BBCH [50] code of Hemp [51], the respective strains were in a different developmental stage at the end of the vegetative phase. Currently publications of *Cannabis sativa* L. [27,52] only distinguish between weeks of vegetative and flowering phase or days after planting but do not give any information on the development of the respective strains at the time of flower induction or final harvest, which limits reproducibility and comparability. Furthermore, no publication is available that reveals the influence of different growing cycles or BBCH [50] stages on yield potential [4].

### 3.2. Assessing the Influence of Light

In order to understand the effect of the different light sources emitting specific light spectra, an understanding of the growth for different strains is a basic prerequisite assessing the possibility to modify or mitigate their final phenology. In addition, there is the possibility of specific interaction of strains with the respective light spectrum as was recently shown in cannabis [27] and soybean [53].

In general, light influences plant photosynthesis and photomorphogenesis, which in turn can affect biomass allocation, yield [6] and as a consequence the complete plant development and growth [39,54]. However, light is only one factor in the cultivation system of *Cannabis sativa* L. and yield and plant growth always reflect an interplay between light, temperature, nutrients and CO_2_ concentration [31]. This makes it difficult to assess real spectral effects. 

Under the conventional ceramic metal halide CHD, the dry mass of the main leaves increased significantly compared to the LED lamps (SOL and AP67) (Figure 5). Regarding the branch leaves, AP67 generated the highest leaf dry matter. The higher biomass allocation of the leaves can be accounted for by the higher radiation temperature under CHD and the generally higher temperatures under AP67 [55]. This tendency was also evident in relation to dry matter of the different stem fractions. No difference between AP67 and CHD in terms of main stem mass, but for branches as there was a significant increase between AP67 and CHD (Figure 7). This behavior has also been characterized by [39], who pointed out that growth under LED lights results in a more horizontal plant development, i.e., a biomass shift from the main to the side shoots at reduced height. In terms of total specific leaf area (Figure 6), CHD was able to increase it significantly, followed by AP67 and SOL. Furthermore, there was a significant difference in maximum photosynthetic capacity (A_max_) between CHD and SOL, but no difference between AP67 and CHD. This finding is consistent with the study of [56] who pointed out that specific leaf area is highly correlated with A_max_ and under CHD the highest values were measured for both. Despite the higher photosynthetic capacity (Figure 8), a significant loss in dry harvest was generated under CHD. This was mainly due to the strain E19 which showed a significant yield loss in the area of the top side buds under CHD (Figure 10). However, this is rather due to the heterogeneity of E19 than to the light spectra. In contrast to [39], no loss of yield was observed under the LED lamps. Additionally, more yield was generated in the side bud fraction under AP67 compared to CHD and SOL (Table 3).

In regards to the growth trajectory, the strains reached a higher maximum height under CHD compared to AP67 and SOL (Figure 3). The other morphological parameters showed significant differences between the individual strains, but these specific traits could not be significantly increased or reduced by the respective light spectra, except for height (Table 1). This elongation of the plants can be attributed to the lowest red: far-red ratio under CHD and the associated shade avoidance reaction [57,58] which increased in the following order SOL < AP67 < CHD. This is also evident under SOL, as there was a tendency towards shorter plants (Figure 3). Our data showed that CHD achieved the highest photosynthetic performance, but this was not reflected in a higher harvest index. Instead, a higher biomass allocation in the area of the main stem concerning the dry masses of the main stem as well as the main leaves was observed. This further underlines the plant’s response due to shade avoidance, which was counterbalanced by a lower allocation in the area of the branches e.g., branch leaves. At the same time there was an elongation of the plant over all strains, which can entail instability. This is also marked in literature as a typical characteristic of high-pressure sodium lamps [38,59,60].

### 3.3. Limitations and Practical Application

The data and results show that the strains have developed their characteristic traits and that these traits were maintained across all light spectra. This supports the assertion that first and foremost the choice of the strain is essential for a successful harvest [4]. This data set can provide the basis for defining breeding-relevant parameters. We recommend breeding for strains with a close ratio between main stem and branches, as E19 with the longest side shoots had the highest yield potential and a significantly increase of side top buds proportion. In addition, a long internode distance on the main stem as well as on the branches is useful to avoid mutual shading of the buds and to allow aeration of the plant stock. With regards to leaf formation, the development of the branch leaves in particular should be completed as soon as possible after the induction of the short day so that a shift towards flower formation with the resulting growth of the sugar leaves can follow.

The height, stem diameter and allocation of the main stem and branches can be positively influenced by the selection of the respective light source. Under the LED lamps, plants were shorter with a higher dry mass of the side shoots under AP67 and no significant loss of yield or reduction of the harvest index. SOL can cause a tendency to reduce the height due to the high R:FR ratio with no significant loss of yield. Furthermore, there were no apparent differences between the light sources and the strains in terms of total yield at DAP 56. This suggests that the last 4 weeks of flowering are crucial to influence the yield structure with light and to assess the yield potential of strains. Additionally, our results highlighted that LED lamps have a more uniform illumination compared to conventional lamps, which is in contrast to statements of [61].

**Figure 11:**
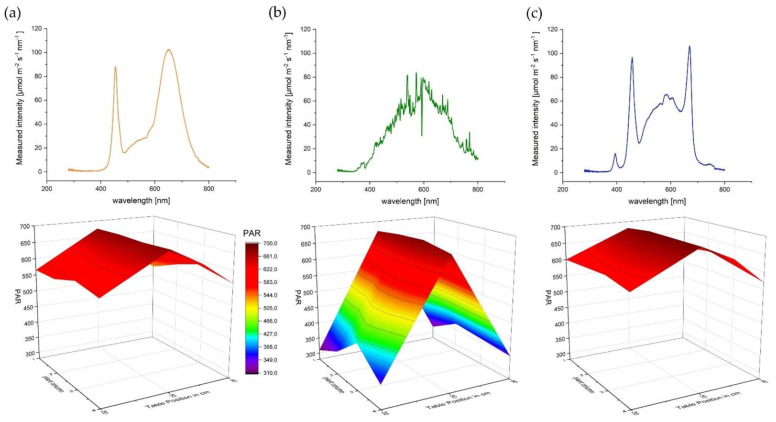
Spectral light intensity [µmol m^−2^ s^−1^ nm^−1^] (top) and distribution of PAR values over the respective plant rows (bottom) for the three light sources (**a**) AP67 (**b**) CHD Agro 400 and (**c**) Solray385^®^.

Nevertheless, a major problem is the comparison and transferability of the results between individual publications on light sources [62]. Since not only different strains and varying lighting strategy (related to the PAR applied) are used, but also different lamps are utilized which have a completely different dispersion (Figure 11). In addition, different environmental conditions such as temperature can influence the results [33] especially when the experiment takes place in a confined space. The most crucial issue here is to establish real repetitions in light experiments as the standard.

## 4. Materials and Methods

### 4.1. Experimental Setup

A greenhouse experiment was carried out at the University of Hohenheim, Germany, between 10 December and 28 March in 2021. Three phytocannabinoid-rich *Cannabis sativa* L. strains A4, Kanada (AI FAME, Wald-Schönengrund, Switzerland) (KAN) and E19(Super Strains, Bladel, The Netherlands) were grown under three light sources: two LEDs namely Solary385^®^ (SOL) and AP67 (Valoya Oy, Helsinki, Finland) and one ceramic metal halide lamp CHD Agro 400 (DH Licht GmbH, Wülfrath, Germany) The light sources are abbreviated asSOL, AP67, and CHD, respectively. Each light source was three times replicated according to a randomized complete block design. Each replicate comprised an area of 1 × 3.5 m. Twelve plants—four per strain- were randomized in a row-column design (3 × 4) within each light source –by-replicate combination. Note that the four plants per strain and replicate resulted in a total of 12 plants. These plants were harvested at four different dates. As the last harvest date was the most interesting one, 6 out of 12 plants were harvested at final harvest, while two were harvested in each of the three harvest dates before. Therefore, six harvest dates were randomized to three replicates each with four plants, and plants from three out of the six harvest dates were harvested at final harvest. Distance between plant rows was 25 cm. All light replicates were separated by black foil to prevent border effects, but at the same time allow enough ventilation assuring a constant microclimate. Greenhouse environmental target conditions were set to 22 °C day temperature and 18 °C night temperature at a humidity level of 60%. These parameters were checked and logged by the greenhouse control system. In addition, Tinytag Plus 2 (Gemini Data Loggers Ltd., Chichester, West Sussex, UK) data loggers were installed in the respective sub-rooms. Resulting environmental conditions throughout the whole trial consisted of night temperatures of 18 °C and mean day temperatures under AP67 of 23.5 °C, SOL 22.4 °C and CHD 22.8 °C respectively. Humidity varied from 40.5 to 80.1% and CO_2_ levels reached between 390 and 450 ppm. Water was provided as needed according to horticultural standard.

### 4.2. Plant Material and Growing Conditions

The experimental plants were propagated from their respective mother plant. From all 240 cuttings (80 per strain), foliage leaves were reduced to 3 leaves per cut and leaf tips were trimmed to reduce transpiration during the rooting phase. The cuttings were treated with Clonex Rooting Gel (city, country, 3.3 g/L indolylbutyric acid) and then placed into 55 mm × 31 mm Eazy Plug^®^ seed cubes (Eazy Plug, HJ Goirle, The Netherlands), which were watered before for 24 h. The cuttings were kept in mini greenhouses under a photoperiod of 18 h with 100 μmol m^−2^ s^−1^ of photosynthetically active radiation (PAR) supplied by the light source AP67. Air humidity was kept between 80–90% by spraying water several times a day. The plants were repotted three times during their growth. After a rooting period of 21 days, the rooted cuttings were transferred to round pots (9 cm), after 18 days to 14 cm and into the final pot size of 29 cm (Lamprecht-Verpackungen GmbH, Göttingen, Germany) during the transition from long to short day. At each repotting 50 g, 230 g and 2050 g of substrate 5 (Klasmann-Deilmann, Geeste, Germany) were added. 15% perlite was added to the peat mixture at each repotting according to [8].

The fertilisation schedule consisted of two different fertilizers. Plantaactiv 18-12-18 Type A was used during the long day and Plantaactive 10-20-30 Type B (Hauert, Grossaffoltern, Switzerland) during the short day period. The fertilizers were applied three times a week. The total amount of fertilizer applied per week is given in Table 4. In addition, 1 g of calcium ammonium nitrate 0-0-27 per plant was applied in week two of the flowering phase. 

### 4.3. Experimental Light Setting

The spectral light intensity was measured with a FLAME-S-XR1-ES spectrometer (Ocean Optics Germany GmbH, Ostfildern, Germany). In order to create equal lighting conditions, light sources were fixed at a height of 1m above the respective table to achieve in the center line 680 μmol m^−2^ s^−1^ PAR The light spectrum and the spectral light intensity for different wavelength ranges are shown in Figure 11 and Table 5. The red (R): far red (FR) ratio was calculated according to [63].

The other two plant rows were then set up in the same way by 25 cm each. The position of each plant was determined at the beginning of the experiment by the statistical design and marked on the table. PAR was measured with a FLAME-S-XR1-ES spectrometer (Ocean Optics Germany GmbH) and checked regularly. Despite the same PAR in the center of each table, a decrease of PAR towards the edge and substantial differences between CHD in comparison to SOL and AP67 were detected (Figure 11). This is typical for each respective light source as they differ in their physical light emission pattern. In order to establish comparability between the different light sources, the PAR values at 1m height above each plant were included in the statistical design as covariate.

### 4.4. Data Collection

#### 4.4.1. Destructive Sampling

**Figure 12:**
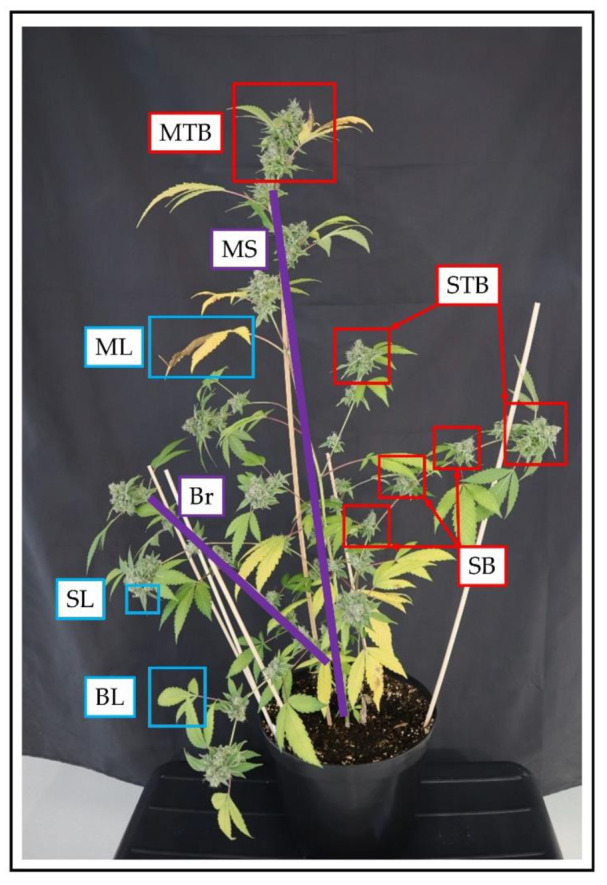
Schematic representation of the fractionation of *Cannabis sativa* L. exemplified by E19 at final harvest. The leaf fraction is divided between main leaves (ML), sugar leaves (SL) and branch leaves (BL). As regards to the stem, it is composed of main stem (MS) and branches (Br). The flowers were divided into main top bud (MTB), side top bud (STB) and side bud (SB).

Plants were destructively sampled three times during the experiment according to Figure 12. Eighteen plants (two plants per strain and light spectrum) were harvested at 28 DAP (end of the long day period), and 56 (after four weeks of flowering) DAP. The final harvest included the remaining 54 plants (six plants per strain and light spectrum).

At each destructive measurement, the plants were cut at the soil surface and separated into main stem and branches. The leaves were detached, the petiole remained at the stem and the leaf area for each fraction (main leaf (ML), branch leaf (BL) and sugar leaf (SL) were measured with an LI-3100 Area Meter (LI-COR, Lincoln, NE, USA). The remaining stem was divided into main stem and branches. Then, the fresh matter of each fraction for stem and leaves was determined and then dried at 70 °C for two days until constant weight and dry matter was recorded. The dry matter of the respective leaf fractions was used to calculate the specific leaf area. Total specific leaf area, obtained using the sum from all specific leaf areas of the individual leaf fractions (main leaves, branch leaves and sugar leaves). For the leaf fraction sugar leaves, the specific leaf area of branch leaves was used and calculated on the basis of the DM of sugar leaves.

The final harvest date was determined when 70% of pistils had darkened [8]. This varied depending on the strains, A4 was harvested after 7 weeks (80 DAP), KAN in the middle of week 8 (85 DAP) and E19 at the end of week 8 (88 DAP) of the short-day period. Harvesting was done in bundles according to light spectra. After harvesting A4, no further fertilizer was applied.

In addition, flower yield per plant was determined for each biomass cut during the short-day period. Flower yield was divided into main top bud (MTB), side top buds (STB), and the remaining flowers (SB). The fresh matter of the trimmed petals (sugar leaves) was also measured. The flowers were air-dried for 14 days at 22 °C and 40% humidity to determine their dry matter and the harvest index (HI). Since different drying methods were used for leaves and stems compared to the flowers, the residual moisture of the flowers was determined for the calculation of the harvest index (HI) and the flower dry matter was adjusted accordingly.

#### 4.4.2. Non-Destructive Measurements

Detailed morphological measurements were carried out non-destructively every three days during the long-day period and every seven days during the short-day period on the 54 plants included in the final harvest. These measurements included plant height [cm] up to the highest node, number of nodes, length of individual internodes, length of the initial branches that emerged from the nodes after propagation, number of branches, number of main leaves and branch leaves. Main leaves were defined as the leaves that develop from the nodes of the main stem. Branch leaves define all remaining leaves (Figure 12).

The monitoring of the height of the highest node, the number of main and reduced leaves was carried out until the final harvest. The diameter of the main stem was measured 2 cm above soil level, i.e., also in the middle of the stem, and an average value was calculated until 70 DAP. Due to the growth of the main bud, the number of nodes from 70 DAP onwards could no longer be assessed. The same applied to the length of the side shoots, as these could no longer be measured due to the growth of the flowers. Simultaneously with the length of the side shoots, the number of side shoots was also terminated.

#### 4.4.3. Photosynthetic Rate

The photosynthetic rate was measured with the LCpro-SD (ADC BioScientific Ltd., Hoddesdon, UK) after week three (49 DAP) and five (63 DAP) of the short day period. To determine the maximum photosynthetic rate (A), the conditions in the measurement chamber of the device were set to 1739 μmol m^−2^ s^−1^ PAR, 30 °C, and an ambient CO_2_-concentration between 399 and 410 ppm. The youngest fully-developed leaf on the main stem was used for the measurement. Values were recorded when a steady photosynthetic rate was achieved. Since the middle leaflet of the *Cannabis sativa* L. leaf does not fill the measuring chamber (2.5 cm × 2.5 cm), the leaf area within the chamber was determined by measuring the diameter at the beginning (BW), in the middle (MW), and at the end (EW) of the chamber after each measurement. The leaf area was then calculated as two trapezoidal surfaces and the measured photosynthetic rate was adjusted by the measurement area by multiplying with the ratio of area/6.25.

### 4.5. Chemical Analysis

To determine the nitrogen concentration of the two leaf fractions, the samples of the second and final biomass cut were homogenized per fraction and plant with a GRINDOMIX GM 200 (Retsch GmbH, Haan, Germany) and then analyzed according to [64] with a thermal conductivity detector Elementar Vario EL (Elementar, Langenselbold, Germany).

### 4.6. Data Analysis

#### 4.6.1. Calculating of Growing Degree Days

To describe the growth and development of *Cannabis sativa* L. a shift from DAP to growing degree days (GGD) is required to include the different microclimate conditions under the respective light treatments in the evaluation.

Growing degree days at day *t* (GDD) equation:

(1) $$\mathrm{GDD}=\sum_{i=1}^{t}\left(\frac{T_{\min_{i}}+T_{\max_{i}}}{2}\right)-T_{\mathrm{base}}$$

The values of $T_{\min_{i}}$ and $T_{\max_{i}}$ were collected the minimum and maximum temperature data of the Tinytag Plus 2 (Gemini Data Loggers Ltd., Chichester, West Sussex, UK) data loggers of the greenhouse system measured at day *i* between planning and the day *t* for which the GDD is to be calculated. $T_{\mathrm{base}}$ was defined as 8 °C according to [65].

#### 4.6.2. Assessing the Growth Trajectory

We used the data from the weekly assessments of the 54 final plants. This dataset consisted of height to the highest node, number of nodes, length of the individual internodes, length of the initial side shoots that emerge from the nodes after propagation, angle of the initial side shoots in relation to the main stem, number of branches, number of main leaves and number of reduced leaves. In a first step, the respective data set was plotted and a trend was worked out. Based on the data trend, a distinction was made between a linear or sigmoidal pattern. Based on the gradients, the following equations were selected:

The classical logistic curve of [66] customised by [67] (2) was selected to determine the growth curve of the maximum plant height:

(2) $${\mathrm{height}}_{tp}=\frac{h_{\max_{p}}}{1+e^{\left(-k_{p}\left({\mathrm{GDD}}_{t}-t_{m_{p}}\right)\right)}}$$

where ${\mathrm{height}}_{tp}$ is the predicted height of plant *p* at day *t*, $h_{\max_{p}}$ is the maximum heipht of plant *p*, ${\mathrm{GDD}}_{t}$ are the growing degree days at time *t* and $k_{p}$ and $t_{m_{p}}$ are plot specific parameters of the growth curve, respectively.

For the parameters number of nodes, length of the individual internodes, length of the initial side shoots that emerge from the nodes after propagation, angle of the initial side shoots in relation to the main stem, number of branches so a linear approach was taken (3):

(3) $$y_{pt}=a_{p}+b_{p}\cdot {\mathrm{GDD}}_{t}$$

where $y_{pt}$ is the observation of one of the traits above of plant *p* at day *t*, $a_{p}$ and $b_{p}$ are the plot-specific intercept and slope of the linear regression.

#### 4.6.3. Statistical Analysis

(4) $$y_{hjklmnp}=\mu +b_{h}+t_{hj}+r_{hjm}+c_{hjn}+\tau_{k}+\varphi_{j}+\rho_{l}+{\left(\tau \varphi \right)}_{kj}+{\left(\tau \rho \right)}_{kl}+{\left(\varphi \rho \right)}_{jl}+{\left(\tau \varphi \rho \right)}_{kjl}+\beta \cdot x_{hjmn}+e_{hjklmnp}$$

where $y_{hjklmnp}$ is the observation of the plant *p* located at the *m*th row, *n*th column on the *j*th table of the *h*th room treated with *k*th strain and *j*th light spectra harvested at *l*th date, μ is the intercept, $b_{h}$, $t_{hj}$, $r_{hjm}$, and $c_{hjn}$ are the random block effects of the *h*th room, *j*th table, *m*th row on *j*th table, *n*th column on *j*th table, respectively, $\tau_{k}$, $\varphi_{j}$, and $\rho_{l}$ are the fixed effects of the *k*th strain, *j*th light spectra and *l*th measuring date, ${\left(\tau \varphi \right)}_{kj}$, ${\left(\tau \rho \right)}_{kl}$, ${\left(\varphi \rho \right)}_{jl}$, and ${\left(\tau \varphi \rho \right)}_{kjl}$ are the fixed two- and three-way interaction effects between the corresponding factors involved, β is the slope of PAR measurement $x_{hjmn}$ at the *m*th row, *n*th column on the *j*th table of the *h*th room and $e_{hjklmnp}$ is the error of $y_{hjklmnp}$. The slope for covariate PAR was dropped in case of non-significance.

Non-destructive measures were taken from plants of final harvest only, thus the model above was simplified by dropping all effects involving date. The reduced model was fitted to all response variables of non-destructive traits including the parameters of fitted curves. Depending on the significance of influencing factors simple or marginal means were fitted. Means were compared using Tukey test and were displayed using a letter display [68]. For means of parameters of fitted plant-specific curves, these means serve as parameters for the for the final curve presented in graphics within the results section. 

In all cases, residuals of fitted models were checked graphically using residual plots. If necessary, data were logarithmically transformed prior to analysis. Afterwards, means were back-transformed for presentation purposes only. Standard errors were back-transformed using the delta method. 

## 5. Conclusions

The study indicated that the tested cannabis strains developed their characteristic morphological growth traits which were maintained across all light treatments. Nevertheless, the height and dry mass allocation to the main stem and branches were positively influenced by the selection of the respective light source. LED lamps generated shorter plants with a higher dry mass of the side shoots under AP67 with no significant loss of yield or reduction of the harvest index. No apparent differences between the light sources and the strains in terms of total flower yield at DAP 56 were observed. Leaf fractions changed over the course of growth and especially the sugar leaves seemed to be of elementary importance in the last growth phase. In addition, the results revealed that the last four weeks of flowering were crucial to influence the yield composition of *Cannabis sativa* L. by light treatments. Choice of cultivar has to be taken into account when interpreting results of light studies, as *C. sativa* subspecies and thus bred strains highly differ in their phenotypic characteristics.

## Figures and Tables

**Table 1:** Estimated parameters of the growth curves for height, number of nodes and mean stem width. k determines the curvature of the growth pattern, and t_m_ is the accumulated amount of growing degree days at which the growth rate reaches its maximum. a is the intercept and b is the slope of the line. Means in one column followed by at least one identical lower case letter did not differ significantly at α = 0.05, within a strain or light spectra as indicated by Tukey-test.

Factor			Height		Number of Nodes		Mean Stem Diameter	
		*hmax*	*k*	*tm*	*a*	*b*	*a*	*b*
		[cm]		[GDD]				
Strain	A4	136.10 ^a^	0.0058 ^a^	410.72 ^a^	8.54 ^a^	0.035 ^a^	3.37 ^a^	0.0034 ^a^
	E19	95.25 ^b^	0.0057 ^a^	347.06 ^b^	7.75 ^b^	0.017 ^b^	3.06 ^b^	0.0027 ^b^
	KAN	66.11 ^c^	0.0045 ^b^	364.65 ^b^	7.96 ^b^	0.019 ^b^	3.06 ^b^	0.0018 ^c^
Light	AP67	94.65 ^b^	0.0053	378.83				
	CHD	114.03 ^a^	0.0053	378.83				
	SOL	87.78 ^b^	0.0053	378.83				
*p*	Light	0.001	0.367	0.737	0.993	0.264	0.117	0.164
	Strain	0.001	0.001	0.001	0.017	0.001	0.009	0.001
	Light × Strain	0.203	0.580	0.408	0.113	0.645	0.421	0.409

**Table 2:** Estimated parameters for the linear regressions of the internode length, branch length and number of branches on GDD. a is the intercept and b is the slope of the line. Means in one column followed by at least one identical lowercase letter did not differ significantly at α = 0.05, within light spectra. Means in one column followed by at least one identical uppercase letter did not differ significantly at α = 0.05, within a strain as indicated by Tukey-test.

Factor			Internode Length		Length Side Branches		No. Branches	
			*a*	*b*	*a*	*b*	*a*	*b*
Light × strain	AP67	A4	1.66 ^Aa^	0.0021 ^b^	0.37	0.017 ^Ba^	−0.88 ^Ba^	0.044 ^Aa^
		E19	1.74 ^Aa^	0.0028 ^a^	0.37	0.048 ^Aa^	1.79 ^Aa^	0.018 ^Bb^
		KAN	1.46 ^Aa^	0.0011 ^c^	0.37	0.020 ^Ba^	2.93 ^Aa^	0.022 ^Ba^
	CHD	A4	1.27 ^Ba^	0.0021 ^b^	0.37	0.007 ^Ba^	−2.90 ^Cb^	0.046 ^Aa^
		E19	1.85 ^Aa^	0.0028 ^a^	0.37	0.028 ^Ab^	−1.00 ^Bb^	0.030 ^Ba^
		KAN	1.46 ^ABa^	0.0011 ^c^	0.37	0.024 ^ABa^	1.72 ^Aa^	0.025 ^Ba^
	SOL	A4	1.65 ^Aa^	0.0021 ^b^	0.37	0.008 ^Ba^	0.10 ^Ba^	0.043 ^Aa^
		E19	1.59 ^ABa^	0.0028 ^a^	0.37	0.043 ^Aa^	2.38 ^Aa^	0.017 ^Bb^
		KAN	1.39 ^Ba^	0.0011 ^c^	0.37	0.020 ^Ba^	2.28 ^Aa^	0.023 ^Ba^
*p*	Light		0.799	0.277	0.436	0.169	0.010	0.028
	Strain		0.001	0.001	0.433	0.001	0.001	0.001
	Light × Strain		0.014	0.101	0.629	0.019	0.016	0.041

**Table 3:** Mean side bud yields in g DM plant^−1^ per light spectra, strain and between the and between the collected biomass cuts at 56 DAP and final harvest (FH). Results are presented as mean values ± standard error (Mean ± SE). Means in one column followed by at least one identical lower case letter did not differ significantly at α = 0.05, within a strain or light spectra as indicated by Tukey-test.

Trait	Light Spectra/Strain	Yield in g DM/Plant
Side bud (SB) light	AP67	3.36 ± 0.23 ^a^
	CHD	2.33 ± 0.24 ^b^
	SOL	2.91 ± 0.23 ^ab^
Side bud (SB) strain	A4	2.33 ± 0.15 ^b^
	E19	3.37 ± 0.30 ^a^
	KAN	2.90 ± 0.12 ^a^
Side bud (SB) date	56	0.92 ± 0.20 ^b^
	FH	4.82 ± 0.15 ^a^
*p*-values side Bud		
Rep	<0.0001	
Light	0.0166	
Strain	0.0013	
Date	<0.0001	

**Table 4:** Fertilization plan per week of key nutrients over the total growth period (DAP in brackets).

	Long Day Period (18/6)				Short Day Period (12/12)							
Nutrients in mg	Week 1 (1–7)	Week 2 (8–14)	Week 3 (15–21)	Week 4 (22–28)	Week 1 (29–35)	Week 2 (36–42)	Week 3 (43–49)	Week 4 (50–56)	Week 5 (57–63)	Week 6 (64–70)	Week 7 (71–77)	Week 8 (78–85)
N	8.1	16.2	32.4	32.4	/	310	60	80	60	40	20	/
P_2_0_5_	5.4	10.8	21.6	21.6	/	80	120	160	120	80	40	/
K_2_O	8.1	16.2	32.4	32.4	/	120	180	240	180	120	60	/

**Table 5:** Spectral distribution of the three light sources for different spectral ranges.

Spectral Range (nm)	AP67	CHD	SOL
PAR 400–700	680	680	680
300–400	2.31	11.00	8.04
400–500	104.50	111.00	121.60
500–600	137.50	264.00	251.49
600–700	438.00	305.00	308.04
700–800	122.76	140.00	34.31
R:FR	4.04	2.83	13.49

## Data Availability

Not applicable.
